# An Unusual Case of Invasive Kaposi's Sarcoma with Primary Effusion Lymphoma in an HIV Positive Patient: Case Report and Literature Review

**DOI:** 10.1155/2015/789616

**Published:** 2015-10-15

**Authors:** Alexandra Millet, Sanmeet Singh, Genelle Gittens-Backus, Kim Ann Dang, Babak Shokrani

**Affiliations:** ^1^Howard University College of Medicine, Washington, DC 20059, USA; ^2^Department of Pathology, Howard University Hospital, Washington, DC 20060, USA

## Abstract

We report a case of AIDS-related Kaposi's sarcoma (KS) with Primary Effusion Lymphoma (PEL) in a 28-year-old, African American male. Kaposi's sarcoma is an AIDS defining disease and typically will disseminate early in the course of the disease affecting the skin, mucous membranes, gastrointestinal tract, lymph nodes, and lungs. This case reports an unusual presentation of the disease along with primary effusion lymphoma. Although the most common organ systems affected by KS are the respiratory and the gastrointestinal systems, the lungs of this patient did not show any evidence of KS. Additionally, the patient demonstrates the rarely seen liver and unique pancreatic involvement by KS along with unusual synchronous bilateral pleural and peritoneal cavity involvement by PEL, adding to the distinct pattern of invasive AIDS-related Kaposi's sarcoma.

## 1. Introduction

Kaposi's sarcoma is a tumor caused by Human Herpesvirus-8 (HHV-8), also known as Kaposi's sarcoma associated herpesvirus (KSHV) [1]. It was originally described in the 1980s by Moritz Kaposi, a Hungarian dermatologist, and has widely become known as one of the AIDS defining diseases [2]. KS is a malignancy of lymphatic endothelial cells that is often highly aggressive in people with HIV and severe immunodeficiency.

Infection of an HIV patient by HHV-8 also provides the setting for the development of another AIDS-related disease, Primary Effusion Lymphoma (PEL). PEL accounts for less than 1–4 percent of all AIDS-related lymphomas. While the disease may occur independently, the overwhelming majority of cases occur in HIV-infected patients. Patients with PEL are often coinfected with HHV-8 and many with Epstein-Barr Virus (EBV) infection as well [3]. The mechanism of the proliferation of the disease with HHV-8 is uncertain.

We report a case of a noncompliant AIDS patient who died from pneumonia and was later found to have an unusual presentation of AIDS-related Kaposi's sarcoma with primary effusion lymphoma.

## 2. Case Presentation

A 28-year-old African American male with a past medical history of HIV/AIDS,* Pneumocystis jiroveci* pneumonia, and latent syphilis presented with shortness of breath, cough, and fever associated with chest pain for the past two weeks. He also complained of nausea, vomiting, and abdominal pain with generalized body swelling. He was noncompliant with his antiretroviral medications and was lost to follow-up with the Center for Infectious Disease Management and Research Clinic.

Upon physical examination, the patient was cachectic in appearance with notable dyspnea. Auscultation of the lungs revealed increased breathing effort with bilateral scattered crepitation. Extremities showed bilateral pedal edema (1+). All other findings were within normal limits.

During the course of the hospital stay, the patient was treated and monitored for pneumocystis pneumonia. He was started on IV antibiotics Bactrim, Zithromax, and Rocephin. He had a CD4 count of 3/*μ*L with a viral load of 73,965 copies. The infectious disease team was consulted and prophylaxis for MAC (*Mycobacterium avium* complex) was recommended. He was also found to have oral candidiasis which was subsequently treated with oral antifungal medication.

**Figure 1:**
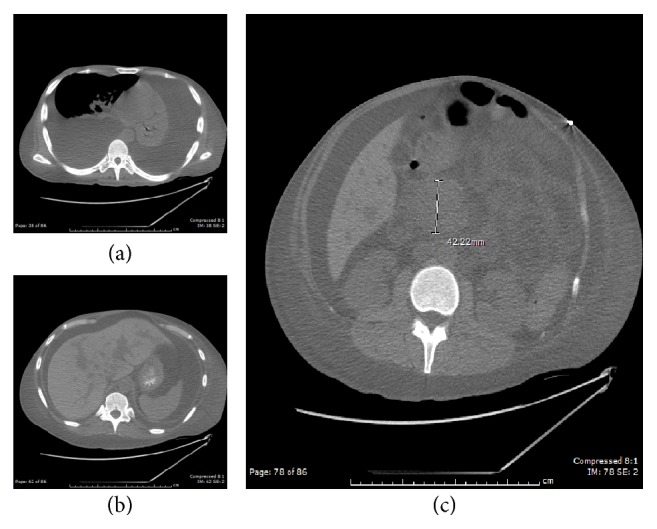
CT scans of the chest and abdomen show large bilateral pleural effusions (a) and severe ascites (b). There is a 4.2 cm mass in the head of the pancreas (c).

**Figure 2:**
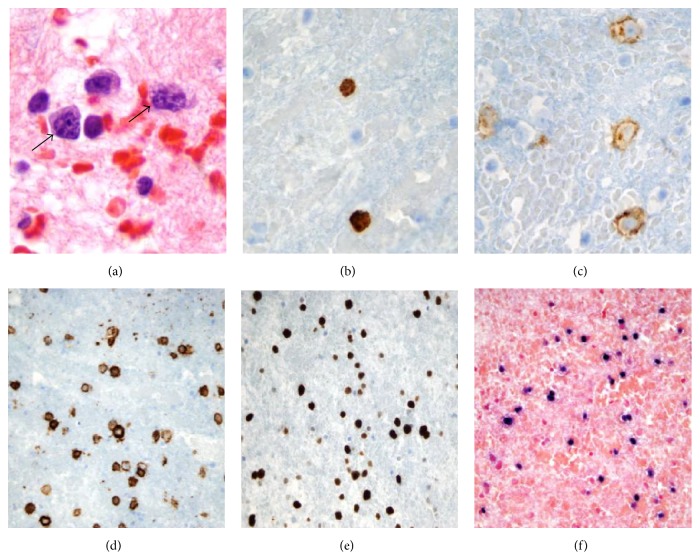
Cytologic evaluation of the pleural fluid shows large atypical lymphoid cells (arrows) ((a) H&E, ×100) expressing HHV8, CD30, and CD138 ((b), (c), and (d), resp.). The atypical cells show high proliferation index highlighted by Ki-67 immunostain (e) and positive staining for Epstein-Barr Virus (EBV) by in situ hybridization (f).

An EGD was performed showing esophageal ulceration and gastroduodenitis. The esophageal biopsy confirmed HSV esophagitis. CT of the chest revealed bilateral pleural effusions, severe ascites, and an enlarged head of the pancreas (Figure 1). Thoracentesis and ascitic taps yielded creamy fluid. Further cytologic evaluation of the pleural and ascitic fluid revealed scattered large atypical lymphoid cells expressing HHV-8, CD30, CD79A, MUM-1, CD56, and CD138 (Figure 2). Atypical cells showed positive staining for Epstein-Barr Virus (EBV) by in situ hybridization. The flow cytometry study on the pleural effusion sample also revealed a monoclonal B-cell population consisting of larger lymphocytes expressing CD45, HLA-DR, CD38, and both surface and cytoplasmic lambda light chain. These findings confirmed the diagnosis of Primary Effusion Lymphoma (PEL).

Subsequent bone marrow biopsy did not show bone marrow involvement. Bilateral chest pigtail tubes were inserted in response to rapidly accumulating pleural effusions.

His septic workup produced a blood culture positive for* Proteus mirabilis*. The patient continued to complain of shortness of breath and later became tachycardic and tachypneic. Following the diagnosis of PEL, Hematology Oncology team was consulted which recommended the chemotherapy with CHOP (cyclophosphamide, doxorubicin, vincristine, and prednisolone) regimen; however, the treatment could not be started since the patient's condition was deteriorated and he developed septic shock associated with severe hypoxemia and hypotension. His respiratory status declined and he was intubated. Later, he was found pulseless with dilated pupils. Cardiopulmonary resuscitation (CPR) efforts were unsuccessful and he was pronounced dead 51 days after admission.

The major autopsy finding in our patient is one of invasive Kaposi's sarcomas involving the feet, the liver, the head of the pancreas, and the gastrointestinal system. Although the lungs are commonly involved in AIDS-associated invasive Kaposi's sarcoma, this patient's lungs did not show any indications of involvement of the disease. Of note, presentation of the disease extending to the liver is rarely reported, and an extensive literature review produced only a few cases of pancreatic involvement.

**Figure 3:**
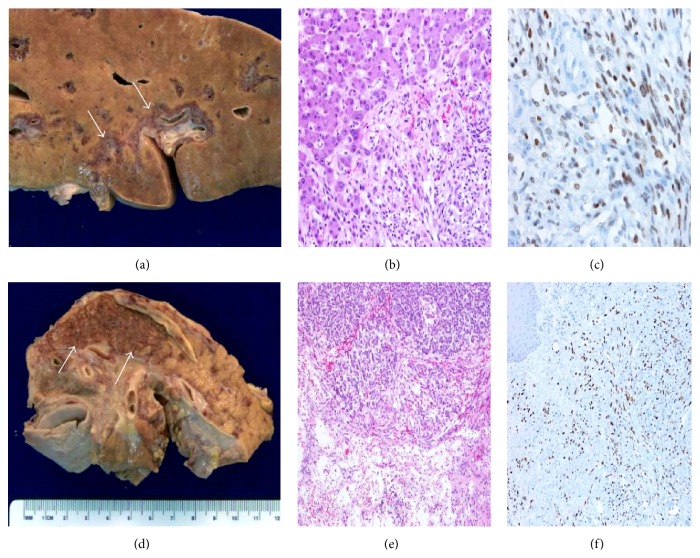
The cut surface of liver shows a well-demarcated perivascular infiltration marked by reddish-brown tumorous tissue (arrows) (a). The head of the pancreas shows a reddish-brown infiltrating lesion (arrows) (d). Representative microscopic sections show spindle cells proliferation involving hepatic lobules ((b) H&E, ×20) and pancreatic acini ((e) H&E, ×10) with positive staining for HHV-8 antibody consistent with Kaposi's sarcoma ((c), (f)).

**Figure 4:**
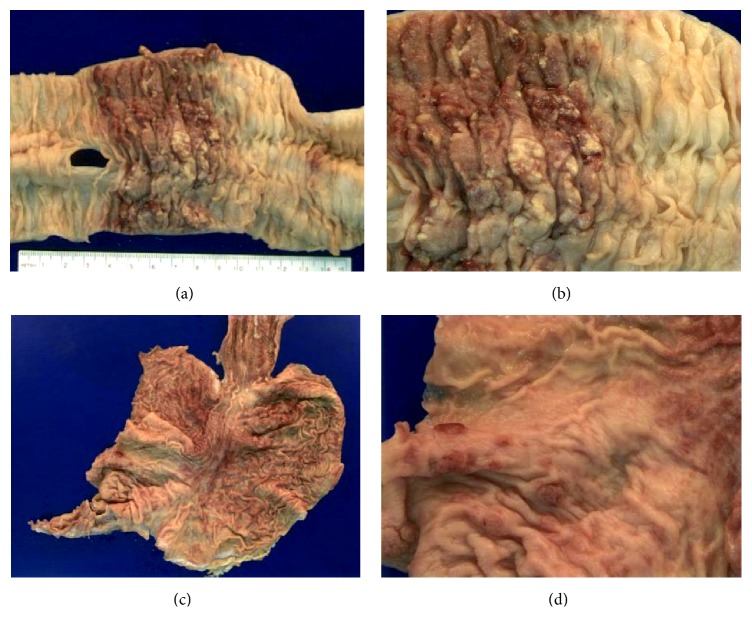
Kaposi's sarcoma with well-circumscribed areas of raised hemorrhagic mucosa in large intestine ((a), (b)) and stomach ((c), (d)).

On gross examination of the patient, coalescing hyperpigmented skin changes on the feet and soles extending to the ankles were noted bilaterally. Tumorous growths were evident in the hepatobiliary system, pancreas, and gastrointestinal tract. The liver showed a well-demarcated perivascular infiltration marked by reddish-brown tumorous tissue (Figure 3). The head of the pancreas showed a 4 × 5 cm reddish-brown infiltrating lesion (Figure 3). The small and large intestine, along with stomach, showed widely separated, well-circumscribed, and circumferential areas of raised hemorrhagic mucosa (Figure 4). The small bowel mesentery was thickened.

In addition, a bilateral serosanguineous pleural effusion and ascitic fluid were present.

Histologically spindle cell proliferation consistent with Kaposi's sarcoma was found in a number of systems. Representative sections from the skin, vertebral bones, small intestine, stomach, colon, mesentery, liver, and the head of pancreas showed areas of interlacing bundles of spindle cells and slit-like vessels along with extravasated RBCs consistent with Kaposi's sarcoma. CD34 and HHV-8 immunostains provided a positive result confirming the diagnosis (Figure 3).

## 3. Discussion

Kaposi's sarcoma is the fourth most common malignancy associated with a viral infection [4].

There are four different types of Kaposi's sarcoma: classic, endemic, posttransplant, and lastly the AIDS-associated or epidemic. AIDS-associated or epidemic KS is the most common cause of tumor development in HIV infected individuals [5]. The manifestations of KS have changed considerably with the advent of highly active antiretroviral therapy (HAART). As a result of HAART, a smaller proportion of KS patients appear to present with visceral disease [6]. In particular, gastrointestinal tract and pulmonary involvement are less frequent among KS-HAART patients [6].

KS is a multifocal tumor that manifests most frequently in mucocutaneous sites, typically the skin of the lower extremities, face, trunk, genitalia, and oropharyngeal mucosa. KS also commonly involves lymph nodes and visceral organs, most notably the respiratory and gastrointestinal tracts [7]. Unusual presentations of KS reported in relation to the gastrointestinal tract involvement include primary KS of the pancreas [7].

Kaposi's sarcoma can be located in the gastrointestinal tract and cause identical symptoms to carcinoma of the same site. Kaposi's sarcoma of the pancreas mimics pancreatic cancer in an HIV-infected patient. Diagnosis can be made by identification of HHV-8 in pancreatic juice or bile, and a successful clinical outcome is possible with intensive antiviral and cytostatic treatment [8].

Our case is unique since the widespread visceral involvement by KS was not diagnosed until postmortem, and multiorgan involvement with symptoms related to each organ was interpreted separately and treated symptomatically.

Additionally the patient presented with an unusual involvement of the pancreas by KS that manifested as a pancreatic head mass, suggesting primary carcinoma of the head of the pancreas.

Primary Effusion Lymphoma (PEL) is a Human Herpesvirus-8 (HHV-8) associated lymphoma localized in body cavities and usually presenting as serous lymphomatous effusions without detectable tumor masses. It typically affects immunocompromised patients and usually involves only one body site, the most common being the pleural cavity; however, involvement of two body cavity sites has been reported in some series [9, 10]. Herein we describe a case of PEL affecting three body cavity sites in an immunocompromised patient.

The majority of PEL cases arise in young or middle-aged homosexual or bisexual males with HIV infection and severe immunodeficiency [11, 12]. The neoplastic cells are positive for HHV-8 in all cases and most cases are coinfected with EBV [13–15]. Patients typically present with effusions in the absence of lymphadenopathy or organomegaly. Morphologically the neoplastic cells exhibit a range of appearance, from large immunoblastic or plasmablastic cells with markedly atypical features including large pleomorphic nuclei which may be lobated, one or more prominent nucleoli, and abundant amphophilic cytoplasm [11, 13].

Body cavity fluid is analyzed cytologically and by flow cytometry for the presence of clonal large neoplastic cells. To be given a diagnosis of PEL, an infection with HHV-8 must be present. A latency-associated nuclear antigen-1 (LANA-1) assay detects any evidence of HHV-8 in tissue samples. Complete blood counts and positron emission tomography/computed tomography (PET/CT) scans should also be performed to determine the extent of the disease [16].

Brimo et al. described a case of PEL in a 69-year-old HIV-negative man, who presented with peritoneal cavity involvement that progressed to involve the pleural and pericardial cavities despite being treated with chemotherapy and valganciclovir. The patient died 5 months following the initial diagnosis [17]. In contrast to our patient, this case was initially a peritoneal cavity disease that progressed to involve the pleural and pericardial spaces despite appropriate treatment.

Our case is unusual in its primary manifestation as synchronous involvement of bilateral pleural cavities and peritoneal space by PEL.

*Learning Points*

1. In the presence of Kaposi's sarcoma in an HIV patient, the possibility of other HHV-8 related tumors such as Primary Effusion Lymphoma (PEL) should be considered.
2. In our case, the patient had rare pancreatic and liver involvement of invasive Kaposi's sarcoma, as well as multiple cavitary involvement of PEL suggesting advanced disease.
